# Agricultural impacts of sustainable water use in the United States

**DOI:** 10.1038/s41598-021-96243-5

**Published:** 2021-09-09

**Authors:** Neal T. Graham, Gokul Iyer, Mohamad I. Hejazi, Son H. Kim, Pralit Patel, Matthew Binsted

**Affiliations:** 1 Joint Global Change Research Institute, Pacific Northwest National Laboratory, College Park, MD USA; 2 King Abdullah Petroleum Studies and Research Center (KAPSARC), Riyadh, 11672 Saudi Arabia

**Keywords:** Environmental impact, Hydrology, Sustainability

## Abstract

Governance measures such as restrictions on groundwater pumping and adjustments to sectoral water pricing have been suggested as response strategies to curtail recent increases in groundwater pumping and enhance sustainable water use. However, little is known about the impacts of such sustainability strategies. We investigate the implications of such measures, with the United States (U.S.) as an example. Using the Global Change Analysis Model (GCAM) with state-level details in the U.S., we find that the combination of these two governance measures can drastically alter agricultural production in the U.S. The Southwest stands to lose upwards of 25% of their total agricultural production, much of which is compensated for by production increases in river basins on the east coast of the U.S. The implementation of future sustainable water governance measures will require additional investments that allow farmers to maximize production while minimizing water withdrawals to avoid potentially detrimental revenue losses.

## Introduction

Despite population increases and economic growth, water withdrawals in the United States (U.S.) have experienced declines over the last decade as efficiency improvements across sectors have lowered excess water losses^1^. However, despite these declines, water demands have not been met with renewable sources of water alone, particularly in the dry regions of the Southwest. Therefore, the pumping of groundwater from deep aquifers has been trending upwards during the same time period^2^. As a result, the share of groundwater to surface water withdrawals increased from 25% in 2010 to nearly 30% in 2015^1^, of which more than half, of the total groundwater, was used for irrigated agriculture^3^. While much of this groundwater is replenished through recharge, several river basins must tap into deep aquifers containing nonrenewable groundwater that can only be replenished over very long time scales^4^.

Nonrenewable groundwater extraction in the U.S. is estimated to have more than doubled since 1960 and is projected to at least double again by the end of the century^5^. Excessive nonrenewable groundwater extraction has several negative impacts to the local environment including land subsidence, water quality degradation, and sea level rise^6–8^. Nevertheless, for many parts of the U.S., nonrenewable groundwater pumping is a necessity. In these areas, nonrenewable groundwater use for irrigation is increasing to meet domestic and international food demands^9–11^. This practice is projected to continue into the future^12, 13^.

In order to address the environmental concerns associated with the increase in nonrenewable groundwater extraction from overly exploited aquifers local governments are beginning to draft and adopt several measures that aim to encourage the sustainable use of groundwater^14–16^. One such measure is the implementation of groundwater governance provisions which call for reductions or complete elimination of nonrenewable groundwater extraction^17–20^. These provisions are often local in nature with focus on county, state or aquifer level restrictions. The Sustainable Groundwater Management Act (SGMA) of 2014 was adopted in California to decrease the groundwater pumping from moderate to highly exploited river basins in the state by 2040. This act calls for basins in which groundwater extraction exceeds replenishment over several years to obtain groundwater sustainability by the 2040 target date^18^. Additionally, the United States Department of Agriculture’s funded project, the Ogallala Aquifer Initiative, was created in 2011 to help reduce water withdrawals and increase the sustainability of agricultural programs in the areas that draw water from the Ogallala Aquifer^20^. Several other state level groundwater provisions currently exist, yet many of them have been around for decades and do not promote defined future goals as specified in the SGMA (e.g., Colorado Groundwater Management Act of 1965; Illinois Groundwater Protection Act of 1987 Alabama Water Resources Act of 1991).

In addition to groundwater pumping measures, altering the price of water for irrigated agriculture has long been argued as a method to further enhance the efficient use of water^21–23^. While changing agricultural water price subsidies often exist to promote sustainable water use, the agricultural sector continues to pay a fraction of what other sectors pay for water, while maintaining large volumes of withdrawals^24^. While true cost comparisons are lacking in the literature, some studies have suggested that the disproportionality results in the agricultural sector paying as little as 1% of the water cost as other sectors^24–26^. Studies have investigated the implications of raising the price of water for irrigation, often finding that as prices increase, farmers are encouraged to invest in highly efficient irrigation practices to require less water while maintaining profit margins^27, 28^.

Despite some local governments implementing sustainability measures for water use and analytical literature pointing to the impacts of localized adoptive measures^29–33^, there is a lack of studies that explore the regional impacts of sustainability measures of this magnitude, especially over century-wide timescales. This study takes the first step towards examining the long-term implications of implementing sustainable groundwater use provisions and alternative levels of water price subsidies for regional irrigated agriculture water withdrawals. We focus on the United States as an example due to the drastic spatial differences in both groundwater pumping and means of meeting agricultural demands (e.g., rain-fed production in much of the east, and significant irrigation in the southwest). The U.S. also offers varying degrees of provisions currently in place regarding groundwater use that can provide comparisons.

For the purposes of this study, we use GCAM-USA, an open-source, community model that represents the interactions among energy, water, agriculture and land use, economy, and climate systems globally with state-level details in the U.S. (Methods). We explore three scenarios with different levels of stringency of nonrenewable groundwater pumping restrictions and water subsidies to irrigated agriculture in the U.S. (Table 1). First, we consider a reference scenario in which assumes a business-as-usual growth, following Shared Socioeconomic Pathway 2 (SSP2) through the end of the century^34^. This scenario assumes that nonrenewable groundwater may be pumped from a basin after water demands exceed an accessible level (Kim et al., 2016) and is available at higher costs. Water price subsidies in the reference scenario are defined as allowing the agricultural water price to be 1% that of all other sectors, explained further below. For each subsequent scenario explored we consider two levels of nonrenewable groundwater pumping restrictions. Complete nonrenewable groundwater restrictions represent an elimination of future pumping, past 2015. Contrarily, no restrictions represent scenarios which follow the reference scenario and allow for water prices to drive demand for nonrenewable groundwater^35^. We consider two levels of water price subsidies to irrigated agriculture (1% and 100%). The 1% assumption represents water prices for the agricultural sector being 1% that of all other sectors, whereas the 100% assumption represents equal cost to all sectors. Finally, for the reference scenario, we assume that renewable water (surface runoff and groundwater recharge) extraction has defined limits dependent upon the amount of water that is deemed accessible^26^ (Methods). If a river basin’s water demands exceed the accessible portion, withdrawals will come from nonrenewable sources and potentially from additional renewable sources at significantly higher cost dependent upon the basin level price of each water source^26^. Availability of nonrenewable water is especially relevant in dry regions where the accessible portion of renewable water is often exceeded within any time period and water must be pumped from nonrenewable sources to meet demands (e.g., California River Basin, Rio Grande River Basin). Additionally, the cross-sectoral price of water is assumed to remain disproportionate as the agricultural sector pay 1% of what all other sectors pay for water. We note that these assumptions are not intended to model explicit governance measures but rather, are meant to be illustrative with the intent of exploring the multisectoral implications of such measures. To that effect, we explore a range of sensitivity cases including those with intermediate assumptions about the levels of stringency of nonrenewable groundwater pumping restrictions and water subsidies to irrigated agriculture (Supplementary Table 1, intermediate scenario results highlighted in Supplementary Figs. 4–6). As we discuss further, we also explore the sensitivity of our results to key modeling assumptions such as climate impacts on water resources and the availability of desalination technologies.

**Table 1:** Scenario naming and component breakdown.

Scenario name^a^	Additional nonrenewable groundwater pumping restrictions^b^	Agricultural water price subsidy^c^	Accessible water^d^ further restricted
Reference	None	1%	No
Sustainable GW	Complete Restriction	1%	Yes
Sustainable GW + no subsidy	Complete Restriction	100%	Yes

^a^Intermediate scenarios are explored in the Supplementary Information.

^b^In addition to the level of water constraints already present in GCAM-USA. Begins in the first future period.

^c^Relative to all other sectors.

^d^Calculated from the hydrologic model Xanthos^37, 38^ and using Turner et al. (2019).

## Results

**Figure 1:**
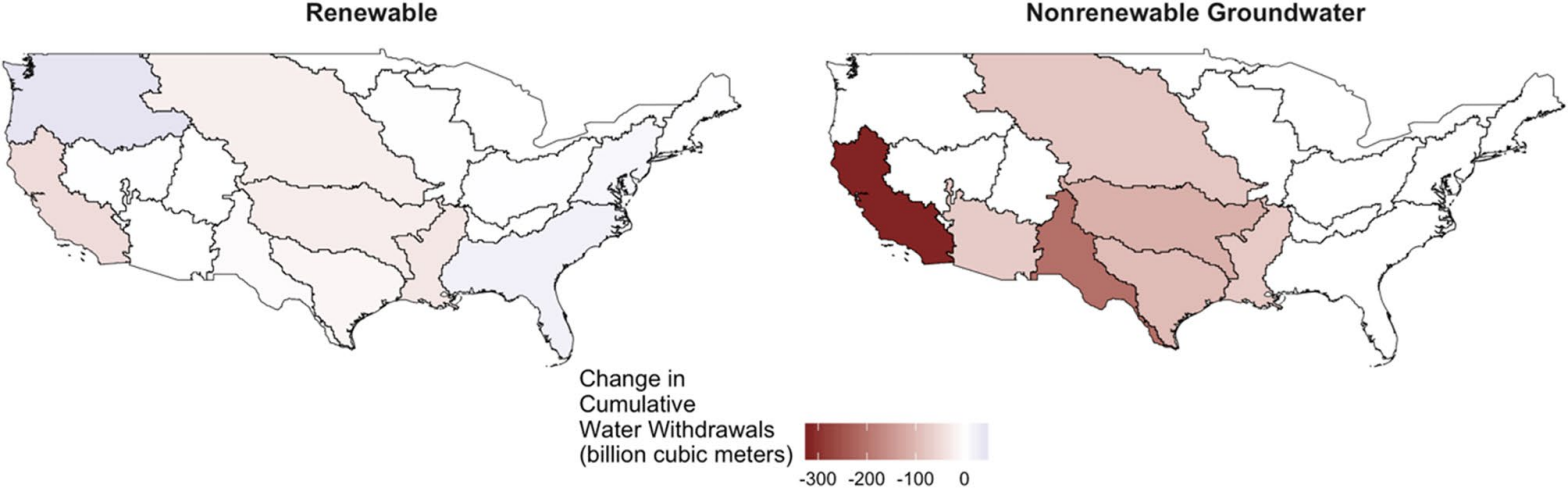
Impact of sustainable water measures on 2015–2100 cumulative water withdrawals (km^3^) by source. Difference between cumulative water withdrawals from renewable and nonrenewable sources from the *Sustainable GW* and *Reference* scenarios. Positive values represent increases in withdrawals after sustainable measures are implemented, whereas negative values represent declines in overall withdrawals when compared to the reference scenario.

The effects of restricting accessible water and nonrenewable groundwater pumping have significant impacts in several river basins around the U.S. as nearly 1000 billion m^3^ of nonrenewable groundwater extraction is projected in the country from 2015 through the end of the century (Supplementary Fig. 1). The implementation of sustainability measures in the *Sustainable GW* scenarios causes this to cease, eliminating all future nonrenewable groundwater extraction (Fig. 1). The California River Basin, much of the Southwest, and Midwest experience the highest impact due to these restrictions as these areas rely heavily on nonrenewable water sources to meet demands. The cumulative loss in groundwater is not offset by increases in renewable water withdrawals as accessible water limits also cause renewable water withdrawal declines in much of the Midwest and in the California River Basin. Therefore, in the *Sustainable GW* scenario, we find that river basins that require nonrenewable groundwater extraction to meet demands will experience compounded renewable and nonrenewable water withdrawal declines as governance measures are implemented. The Pacific Northwest and South Atlantic Gulf basins are the only areas which observe increases in renewable withdrawals as the total volume of accessible water is not withdrawn under *Reference* scenario conditions. It is important to note that the level of water withdrawal changes depicted here represent the upper bounds of outcomes across all intermediate scenarios highlighted in the Supplementary Material.

**Figure 2:**
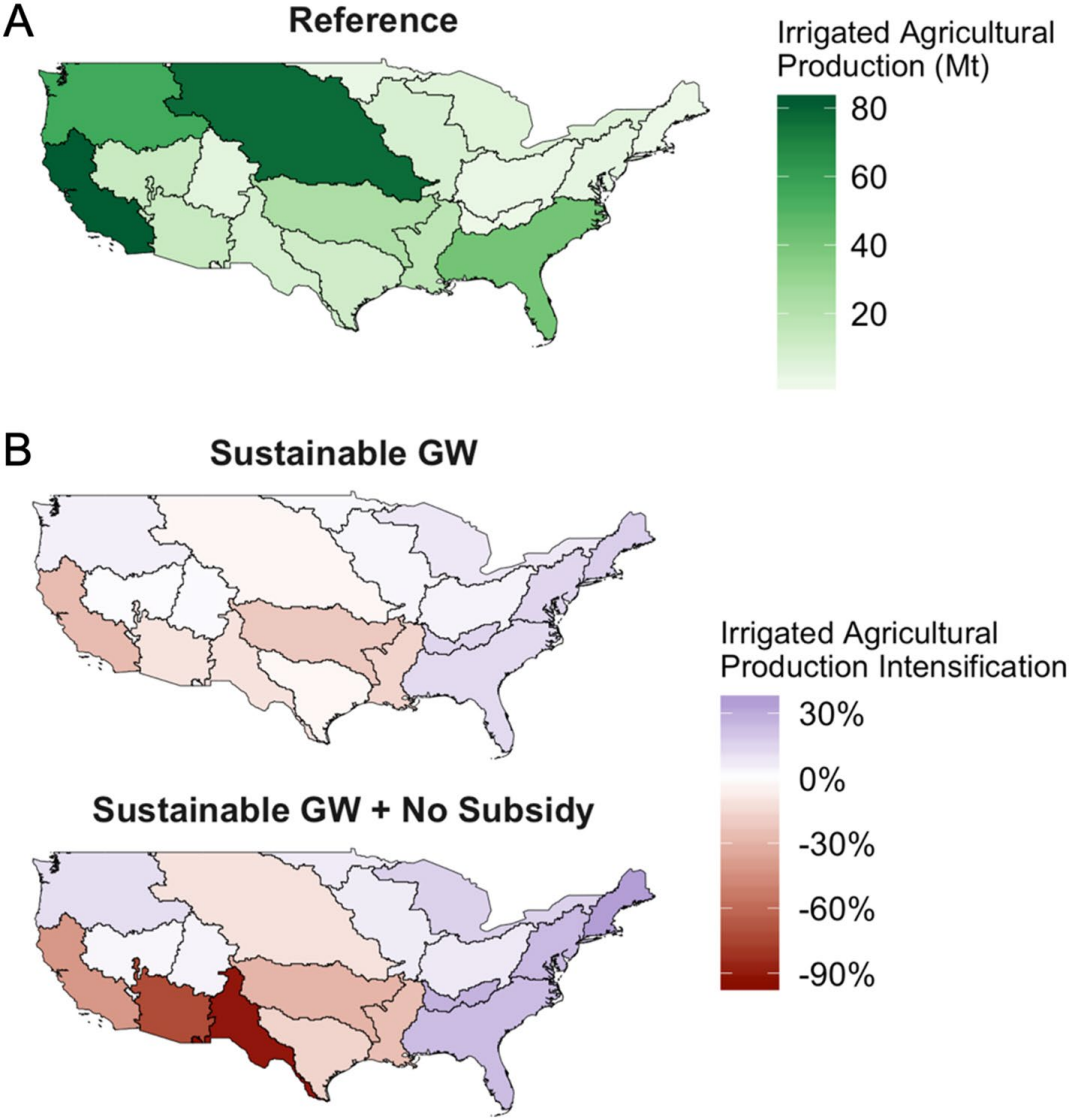
Impacts to end of century irrigated agricultural production across two extremes of future water use. **(A)** Irrigated agricultural production in 2100 for the Reference scenario. (**B)** Relative agricultural production losses and gains in 2100 in the two test scenarios.

Agricultural production is impacted due to declines in nonrenewable groundwater extraction and shifts in withdrawals across basins (Fig. 2). In the absence of sustainable water use measures, irrigated agriculture by end of century is projected to be concentrated in the Missouri River Basin, California River Basin, and the Pacific Northwest, similar, yet intensified from 2010 values (Supplementary Fig. 2), due to large volumes of both renewable and nonrenewable water pumping (Fig. 2A). However, with the addition of either of the water sustainability measures, irrigated agricultural production undergoes spatial shifts around the U.S. as a lack of water availability forces intraregional production compensation. In the *Sustainable GW* scenario, the California River basin and the Arkansas White-Red basins experience the most significant production losses in 2100, with losses approaching 25% of the total basin-level production in the *Reference* scenario (Fig. 2B, top). In the *Sustainable GW* + *No Subsidy* scenario (Fig. 2B, bottom), the impacts are further exacerbated as the Rio Grande River basin experiences nearly a complete loss of irrigated agriculture. In addition, the Lower Colorado River basin loses more than half of their production and the California River basin loses nearly 50% of the agricultural production in 2100 as in the reference scenario. Finally, the spatial variability of changes under the *Sustainable GW* + *No Subsidy* scenario is to depend highly on the level of climatic impact on water availability and the overall access to desalinated water for agriculture (Supplementary Fig. 6). The loss of irrigated agricultural production in the Southwest is partially offset by increases in rainfed production in the eastern U.S. and the Pacific Northwest (Supplementary Fig. 3). However, much of the production losses are compensated for along the east coast of the United States. Each river basin along the Atlantic Ocean coast experiences at least a 25% increase in annual irrigated agricultural production to offset losses brought upon in *Sustainable GW* + *No Subsidy* scenario. On the net, the total production in the U.S. in both sustainability scenarios remains lower than that in the *Reference* scenario, resulting in a minor increase in net agricultural imports into the U.S.

**Figure 3:**
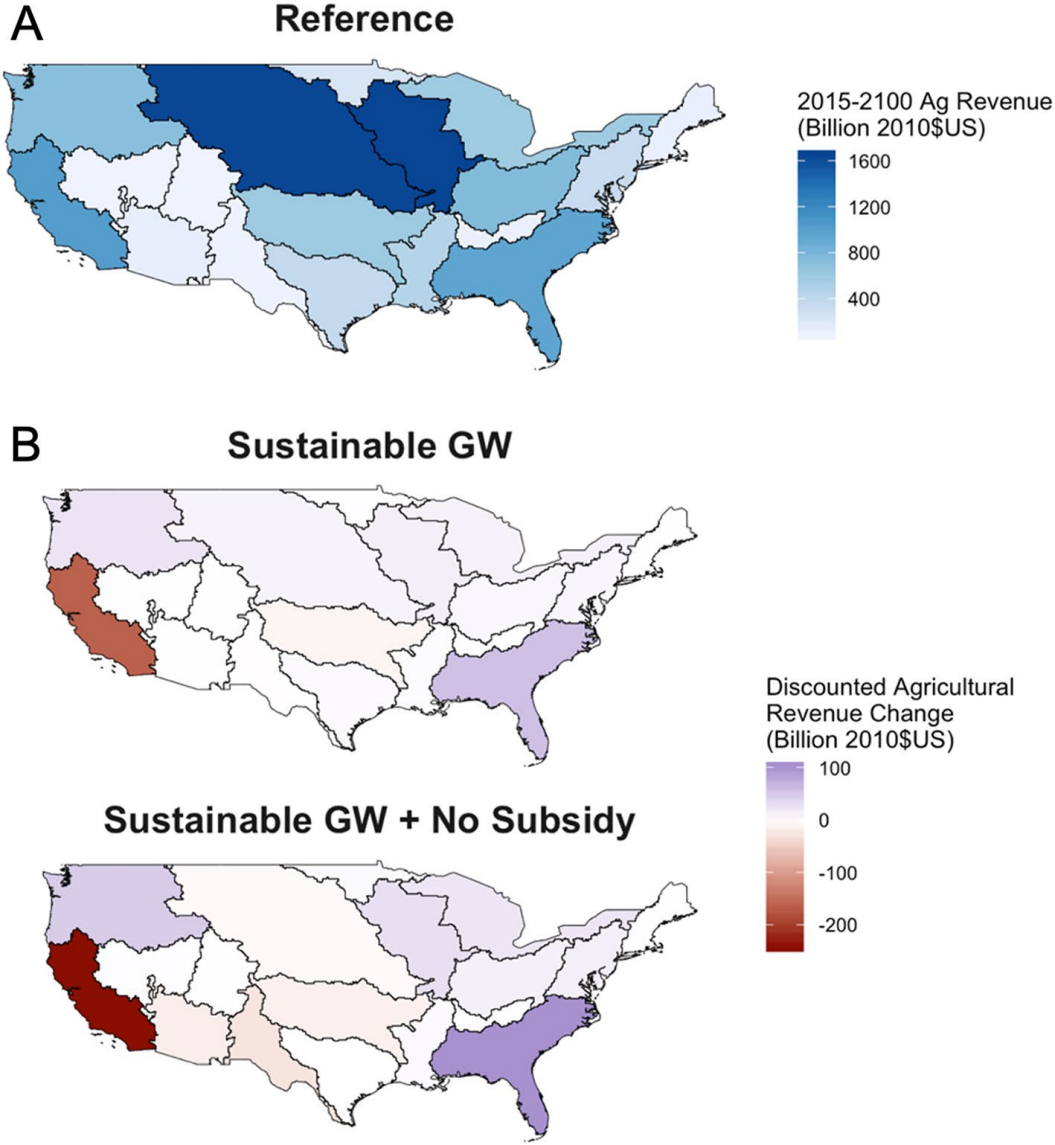
Economic Impact of differing sustainable futures. **(A)** Discounted agricultural revenue in the Reference scenario for the period 2015–2100 (Billion 2010$US). (**B)** Change in discounted agricultural revenue for the 2015–2100 time period across the two test scenarios. When the most stringent sustainability measures are taken, the California River basin experiences nearly 25% total revenue loss.

The agricultural sector contributed more than 5% to the U.S. GDP in 2017^39^, with individual states having much larger shares within their respective economies. Therefore, changes in the agricultural production will result in important economic impacts throughout the U.S. (Fig. 3). For example, the California River basin experiences revenue losses similar to production losses (Fig. 2), reaching nearly 20% total revenue loss in the Sustainable GW + No Subsidy scenario. While the Rio Grande basin experienced a nearly complete production loss in the most extreme scenario, the total agricultural revenue in the basin, under reference conditions, is much less than the rest of the U.S. basins. In the California River basin, losses approach or exceed $50 billion U.S. dollars in all complete sustainable groundwater scenarios (Supplementary Fig. 4). While the above river basins experience losses, others respond with gains. The Pacific Northwest and South Atlantic Gulf basins are two of the largest increases, as these river basins currently represent a large portion of irrigated agriculture outside of the Midwest and California (Fig. 2, top). In total, the U.S. experiences a loss in revenue under both the *Sustainable GW* and *Sustainable GW* + *No Subsidy* scenarios as declines in agricultural production result in a need for increased imports and a loss of profitability in the U.S. agricultural system (Supplementary Fig. 4).

While the results expressed within this study highlight the upper bounds of certain water sustainability measures, there are several intermediate scenarios where future individual governance policies may align. These scenarios are presented in the Supplementary Information for further reference. Independent of scenario assumption, every sustainable water use future includes a decrease in overall agricultural revenue in the U.S. through 2100 (Supplementary Fig. 4). Revenue losses increase significantly once nonrenewable groundwater restriction pass 50% and further increase once the water price subsidy approaches 50% (Supplementary Fig. 4 and 5). Several basins are found to be particularly vulnerable to the complete restriction of nonrenewable groundwater pumping with revenues drastically dropping in the Lower Colorado, Rio Grande, and Arkansas White Red basins due to a drastic reduction in their ability to grow irrigated crops (Supplementary Figs. 6, 9, and 13).

The results expressed so far are contingent on the model, water availability assumptions and level of agricultural price subsidy (“Methods”). In particular, our analysis has assumed no climate impacts on renewable water availability and an exclusion of seawater desalination. Both assumptions allow for the possibility of altered spatial and temporal water availability. For example, climate impacts on renewable water availability have the potential to either exacerbate or ameliorate water stresses within a region, causing a resultant price response for water resources. Likewise, the availability of desalination may allow for additional water in areas with seawater desalination capabilities, which in turn would decrease pressures on other sources of water. To test the sensitivity of our results to these assumptions, we applied future climate derived water availability scenarios from five general circulation models (GCMs) for the Representative Concentration Pathway (RCP) 6.0 W/m^2^ climate future and the ability to invest in seawater desalination in coastal states (Methods). We find that certain GCMs project increased spatial variability of water availability due to climatic changes which, in turn, could ameliorate some of the losses by 2100. For example, in river basins such as the California River, the *Sustainable GW* scenarios experience large variations in economic impacts under varying price subsidies. Total impacts vary from ± 50% of the mean impact, highlighting both the sensitivity to model projected water availability and the potential economic impacts of such futures (Supplementary Fig. 5). These results are spatially variable as several river basins, particularly ones that rely heavily on rainfed agricultural production, experience little impacts as a result of the addition of climate derived water availability and seawater desalination. The sensitivity of future nationwide economic agricultural revenues to these assumptions is rather modest (1%-5% across all sensitivity cases under the *Sustainable GW* + *No Subsidy* scenario, see Supplementary Fig. 5 and 6). This emphasizes that the climatic and desalination impact is likely to be local rather than country wide (sensitivity analysis parameters are summarized in Supplementary Table 1).

## Discussion

The sustainable use of water is becoming increasingly important as socioeconomic drivers lead to increases in water demand around the world. As this demand exceeds renewable thresholds, countries are increasing their depletion of nonrenewable groundwater, which results in significant increases in unsustainable utilizations of water. However, little is known about future socioenvironmental implications of increasing water sustainability in the United States. This study has taken a first step in the investigation of impacts driven by the creation of a sustainable water initiative in the United States by setting limitations on renewable water availability, implementing sustainable groundwater provisions, and changing agricultural water price subsidies.

We have explored the implications of scenarios that mimic a wide range of potential real-world governance measures to show that the adoption of sustainable groundwater usage measures could significantly impact agricultural revenue in the Southwestern U.S. (Supplementary Fig. 4), while concurrently vastly improving the sustainable water use within the U.S. With agricultural revenue constituting a large portion of states’ and the regional economy, such losses can have lasting impacts without additional measures set to maximize water use efficiency. It can be implied that agricultural efficiency improvements designed to better utilize water and maintain maximum crop production could be important to reduce revenue losses from the agricultural sector as available renewable water, particularly in dry years, may be insufficient to meet demands.

The governance measures considered in this paper may impact non-agricultural sectors differently. For instance, the fuel mixes in the power sector are largely unaffected because water for electricity becomes less binding in the future (Supplementary Figs. 7 and 8). Shifts to gas powered power plants, retirement of coal power plants, and the adoption of increasingly renewable power sources allow for water demands to significantly decrease in the United States towards the end of the century. However, some future biofuels rely directly on irrigated agriculture to meet demands for such derivatives as corn ethanol. Sustainable water measures were shown to shift production across basins (Fig. 2), which include biofuel sources moving out of the central basins in the United States (Supplementary Figs. 9–13). This shift also results in decreased total regional production of these crops, driving an increased interregional trade dependency (Supplementary Fig. 5).

Agricultural water price adjustments have been shown to incentivize the adoption of efficient irrigation technologies^21–23^. Our results suggest that in the absence of such measures, dry agricultural regions with high reliance on irrigation might face severe agricultural revenue losses as production shifts elsewhere. For example, in states such as Arizona and California, where nearly 100% of the agriculture is irrigated, altering the subsidized price of water above a certain threshold may cause significant revenue losses (Supplementary Fig. 4–6). These results highlight the sensitivity of intersectoral water price differences particularly as it relates to the agricultural sector. While paying a higher price for water results in overall declines in water usage, addressing the economic impacts of such losses will need to be considered under any future water governance measures.

Our results suggest broader qualitative implications for the global agricultural system. For example, depending on the degree to which states, countries, and regions cooperate on sustainability measures, production could shift to regions which have significantly more renewable water at lower costs or to countries which do not introduce sustainability measures. This movement towards sustainability can shift the global agricultural trade market as countries, particularly those with significant nonrenewable groundwater extraction levels such as the Middle Eastern countries, begin to incorporate sustainability measures. The level of trade and the ability to incorporate necessary interregional trade changes in response to sustainability measures will drive the ability to adopt measures that significantly reduce the unsustainable use of water.

This study provides a first step analysis in how the U.S. may respond to varying degrees of water sustainability governance measures in a multisector modeling framework. We have included the ability to switch between different sources of water dependent on price and availability, and while there is little impact to the energy sector, we have actively included these cross-sectoral feedbacks. Regardless, we find that the agricultural sector will be most impacted by the sustainable use of groundwater and variable sectoral water pricing in the future. Increased accounting of climatic futures and climatic impacts to the agricultural system, including but not limited to changes in evapotranspiration, yield, and water use requirements would expand the understanding of sustainable futures in combination with climatic changes. Further comprehensive economic analyses could shed light on the economy-wide implications of sustainable water use measures.

## Materials and methods

### Overall description

This analysis uses GCAM-USA, of the Global Change Analysis Model (GCAM) version 5.2, to investigate the impacts of limiting available renewable water to the calculated accessible portion, reducing the amount of allowed nonrenewable groundwater extraction, and varying the water price subsidy on the agricultural sector in the United States. By default, each of the 235 river basins in GCAM are limited to 25% of the available total nonrenewable groundwater stock^35^. This value is then limited by up to 100% more dependent upon scenario (Table 1 and Supplementary Table 1), in each of the major river basins of the United States. In addition, GCAM-USA uses a default price subsidy on agricultural water withdrawal prices of 1% of all other sectors for all regions. This number is then linearly increased through 2100 (Table 1), up to values of equal price (100%) of all other sectors. The impacts on water withdrawals are analyzed for each of the basins in the United States. Additionally, the impacts on agricultural production and revenue are analyzed across the United States. Below we describe the GCAM-USA model and scenario components.

### GCAM-USA

GCAM-USA is a market-equilibrium model and submodule of GCAM^40–42^, that links energy, water, land, economy, and climate systems. GCAM-USA includes state level representations of supply and demand for the electricity, industrial, and municipal sectors^43–45^. Also included are agricultural supply and demands at the HUC-2 basin level scale (Hydrologic Unit Codes^46^) same resolution as GCAM’s 235 river basins) within the United States, and livestock and primary energy mining modeled at the United States regional level. GCAM-USA adjusts prices of goods and services within each model time step to equilibrate the supply and demand of goods and services at each time step, and thus simultaneously clears markets across sectors.

GCAM-USA models agricultural production and demands at the HUC-2 basin level with land use competition following a logit-model of sharing driven by the profitability of competing uses of land. This sharing allows for the switching of land use types driven by profitability, with some restrictions dependent upon defined land use nodes. Excess agricultural production from within basins in the United States are then allowed to be traded globally. Any additional imports required for the U.S. would come from the global agricultural market dependent upon each of the other 32 regions within the global GCAM and their individual exports.

GCAM-USA includes water demands for each of the previously mentioned sectors modeled at either the state, basin, or national level^45^ (This Study). For sectors which are not modeled at the state level, water demands are mapped to individual states following an aggregation of historical grid-level water demand data^36^. Water supply in GCAM-USA is modeled at the 235 global HUC-2 basin scale^26, 35^. This study accounts for a limited supply of water by employing cost resource curves across all river basins that follow a logit formulation to determine the share of each water source (renewable, nonrenewable, and desalinated water) needed to meet the water demands within all river basins. As depletion of various water sources increases, the extraction price increases, which leads to corresponding price increases in the goods and services that require higher-priced water sources.

### Key scenario components

#### Accessible water restrictions

Within each river basin of GCAM-USA, an amount of renewable water has been deemed accessible for human use at low cost. This is calculated using the global hydrologic model *Xanthos*^37, 38^ which calculates the percentage of runoff that is stable in dry years while requiring 10% of streamflow maintained for environmental purposes^26, 35^. If groundwater extraction occurs, the accessible portion of water is back-calculated from the total water supply and water withdrawals in the historical calibration period of GCAM-USA. Within the Reference scenario, river basins are allowed to draw more than the accessible portion of water at an increasingly higher cost, which is assumed to include the potential costs of river rerouting, dam construction, or transportation, which are not explicitly captured in GCAM-USA. As the accessible portion is reached, a water price interaction between nonrenewable groundwater and renewable water begins in which basins withdrawal the cheapest source of water. For each unit of water that is further withdrawn causes price increases to account for previously mentioned interventions for renewable water and increased pumping costs for deep groundwater Within this study, we have set a cap on the amount of water that can be withdrawn by using (1.05 * accessible portion) in order to limit total extraction to just above the calculated value. This cap replaces the current cap, or 100% extraction, in the resource cost curve at extremely high cost.

#### Groundwater constraints

Nonrenewable groundwater extraction for GCAM-USA is defined by increasingly expensive cost grades which cause the cost of pumping water to increase as the nonrenewable resource is exploited^35, 47^. For each scenario with additional groundwater constraints in this study, reductions are introduced in 2015 from the total stock on groundwater availability^35^ for each river basin. This causes a decline in the available water grades up until there is a complete elimination of future nonrenewable groundwater pumping allowance in the Sustainable GW scenarios, beginning in 2015.

#### Price subsidies

Producers in the agricultural sector have historically paid less for water than producers from all other sectors. The current assumption in GCAM-USA is that the agricultural sector will pay 1% the cost of water as all other sectors^26^. This can be varied to increase or decrease the level of subsidy that is desired. For this study we implement a linear interpolation from a 2010 value of 1% subsidy to a final 2100 value determined by the scenario. All non-agricultural sectors pay the total cost of water (e.g., 100%) for their respective basin in each scenario.

#### GCM derived climate impacts

For the sensitivity analysis preformed in this study, we include climatic impacts on water supply. These impacts are derived from five different general circulation models (GCM). We calculate the impact by using downscaled and bias-corrected climate data from the Inter-Sectoral Impact Model Intercomparison Project (ISI-MIP)^48^. The global hydrologic model Xanthos^37, 38^ calculates climate derived changes to renewable water supply, using the Penmen Monteith evapotranspiration equation, for the 235 global river basins using necessary GCM outputs. GCM derived rainfall and temperature at the grid scale is used to calculate renewable water supply and then aggregated to the 235 GCAM basin scale.

## Supplementary Information

Supplementary Information.
