# Supplementary material for: Agricultural impacts of sustainable water use in the United States
Source: Sci Rep. 2021 Sep 9;11:17917. doi: 10.1038/s41598-021-96243-5 (PMC8429431; doi:10.1038/s41598-021-96243-5)
Supplement: Supplementary file 1 — Supplementary Information. [file 41598_2021_96243_MOESM1_ESM.docx]

**Supplementary Information for:**

**Agricultural impacts of sustainable water use in the United States**

Neal T. Graham, Gokul Iyer, Mohamad I. Hejazi, Son H. Kim, Pralit Patel, Matthew Binsted

**Supplementary Information**

**Calculations**

*Discounted Agricultural Revenue*

This study uses the net present value calculation in order to evaluate the discounted agricultural revenue, at 5%, in each river basin for the 2015-2100 time period (Equation 1). This calculation is used in the formulation of Figure 3 and Supplementary Figs 4 & 5. Here b represents values for any GCAM HUC-2 basin, i represents model year, j represents individual scenarios, Q represents agricultural production (Mt/year) and P is commodity price (USD/Mt).

|  | ${\Delta NPV}_{b}= \sum_{i=2015}^{2100} \frac{\left( P_{i,j}* Q_{i,j} \right)-\left( P_{i,ref}* Q_{i,ref} \right)}{\left( 1+r \right)^{\left( i-2015 \right)}}$ | (1) |
| --- | --- | --- |

**Supplementary Table 1:** Sensitivity analysis parameters

| Parameter | Degree of Implementation |
| --- | --- |
| Nonrenewable Groundwater Pumping Restriction | 1. No restriction 2. 25% reduction 3. 50% reduction 4. 75% reduction 5. Complete restriction |
| Agricultural Price Subsidy | 1. 1% 2. 5% 3. 10% 4. 50% 5. 100% |
| Climatic Impacts | 1. Consistent Water Availability 2. RCP 6.0 derived water availability from 5 GCMs (Methods) |
| Seawater Desalination Availability | 1. None 2. Full Availability in coastal basins and states |


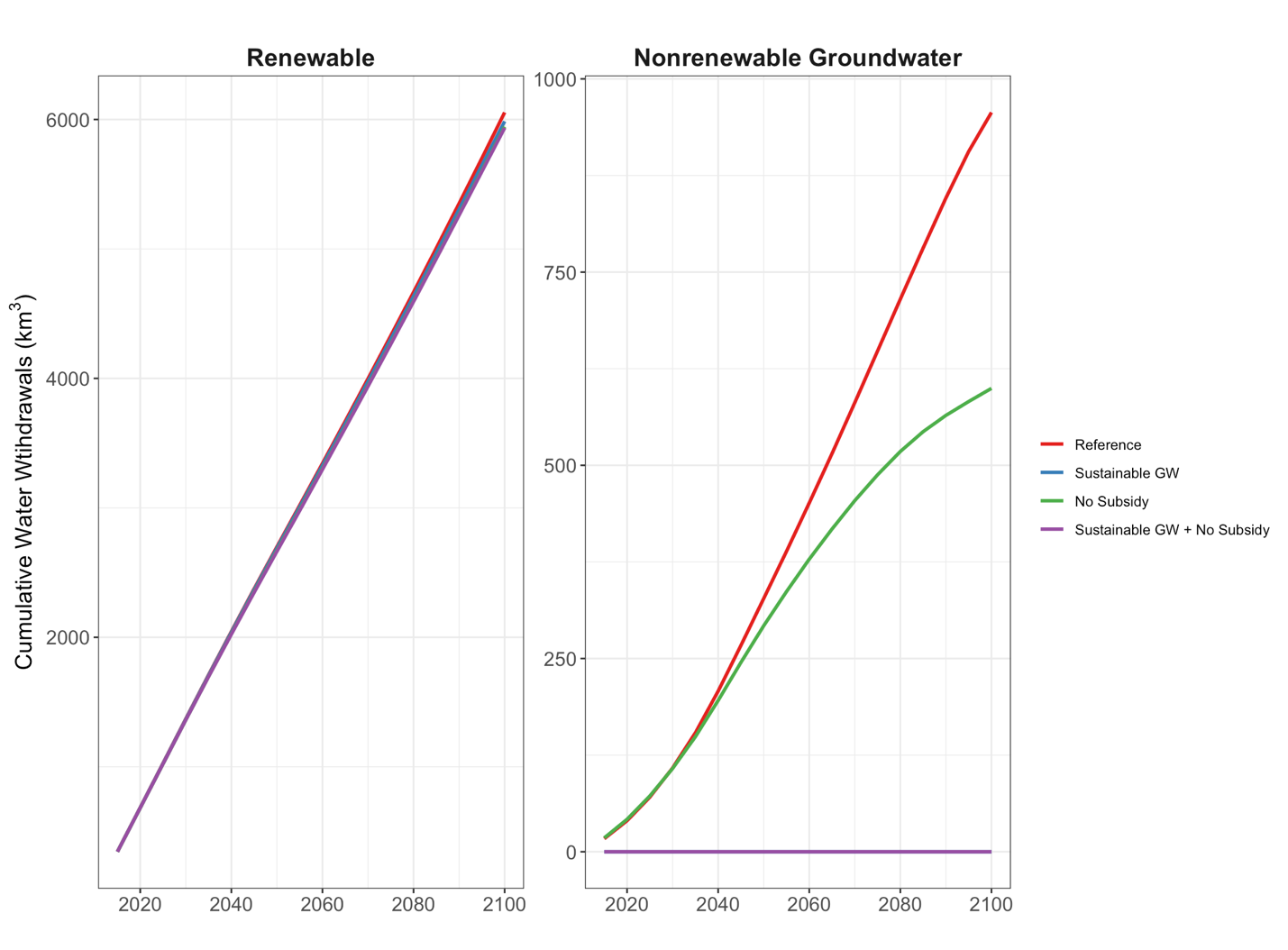


**Supplementary Figure 1. Cumulative renewable and nonrenewable water withdrawals for the USA region.** **A,** Renewable water withdrawals (billion m^3^) across all scenario for the period 2015-2100. **B,** Nonrenewable water withdrawals (billion m^3^) across all scenarios for the period 2015-2100.


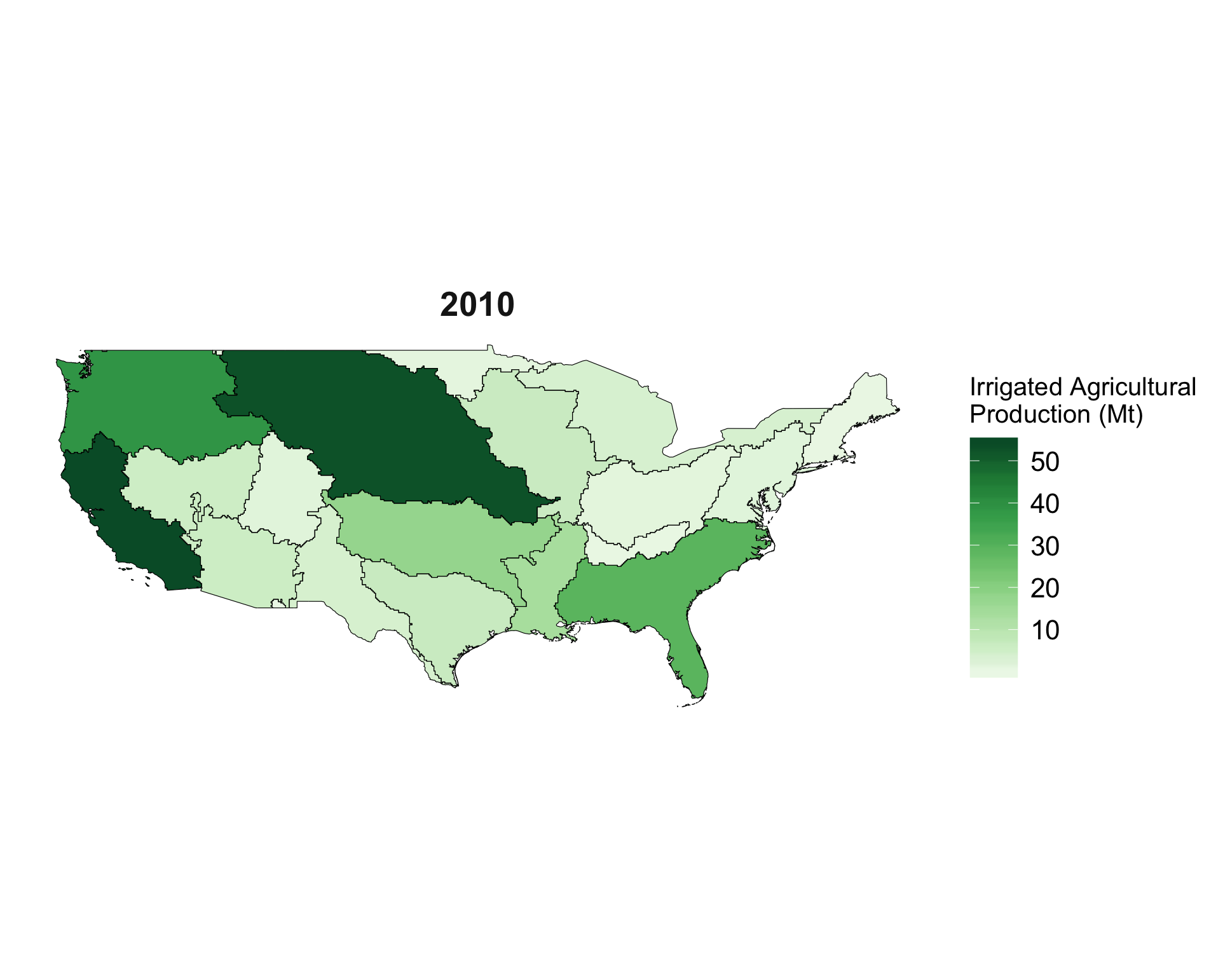


**Supplementary Figure 2 Agricultural Production in 2010 for HUC-2 basins in the United States (Mt)**


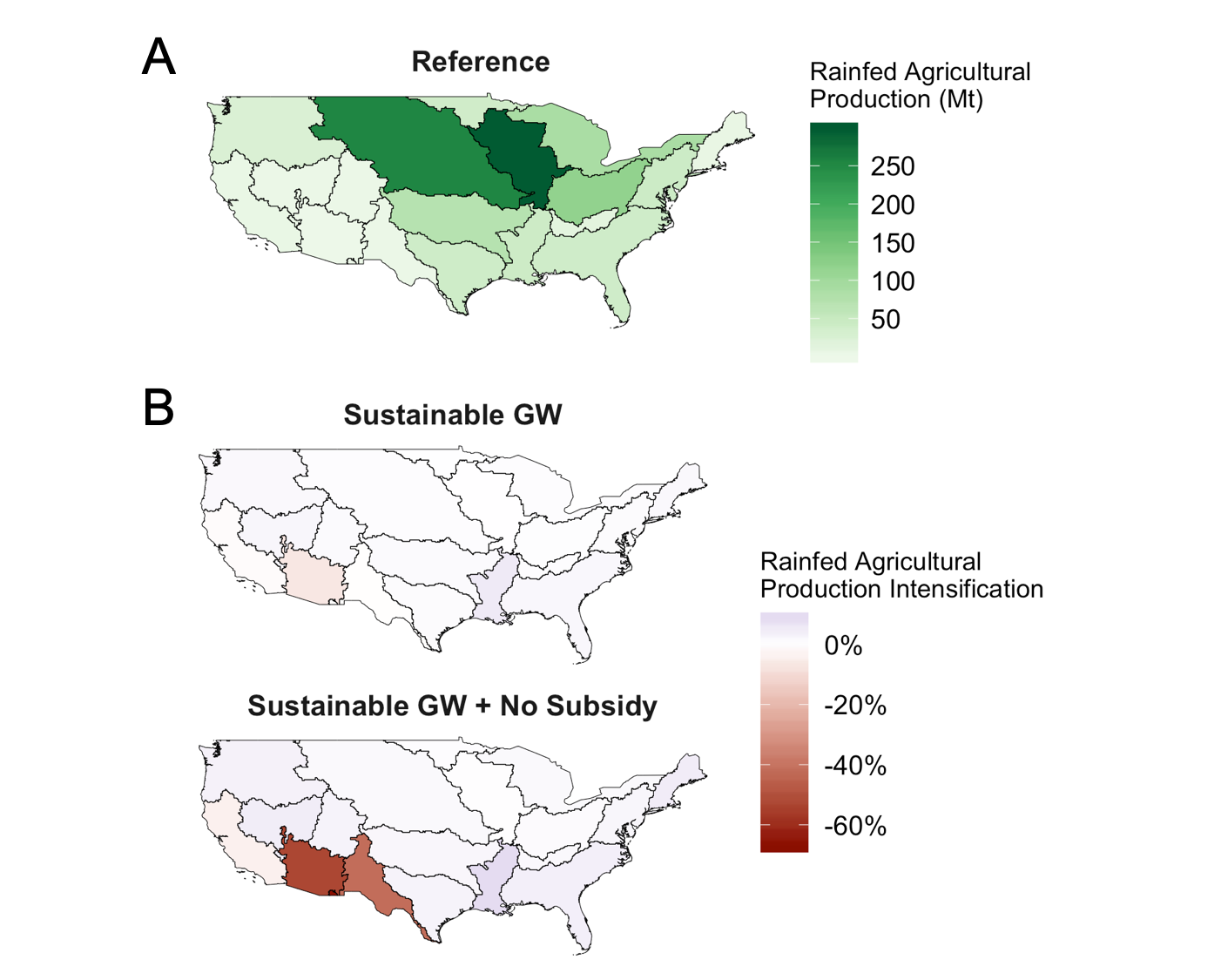


**Supplementary Figure 3. Impacts to end of century rainfed agricultural production across two extremes of future water use.** **A,** Rainfed agricultural production in 2100 for the Reference scenario. **B,** Relative agricultural production losses and gains in 2100 in the two test scenarios.


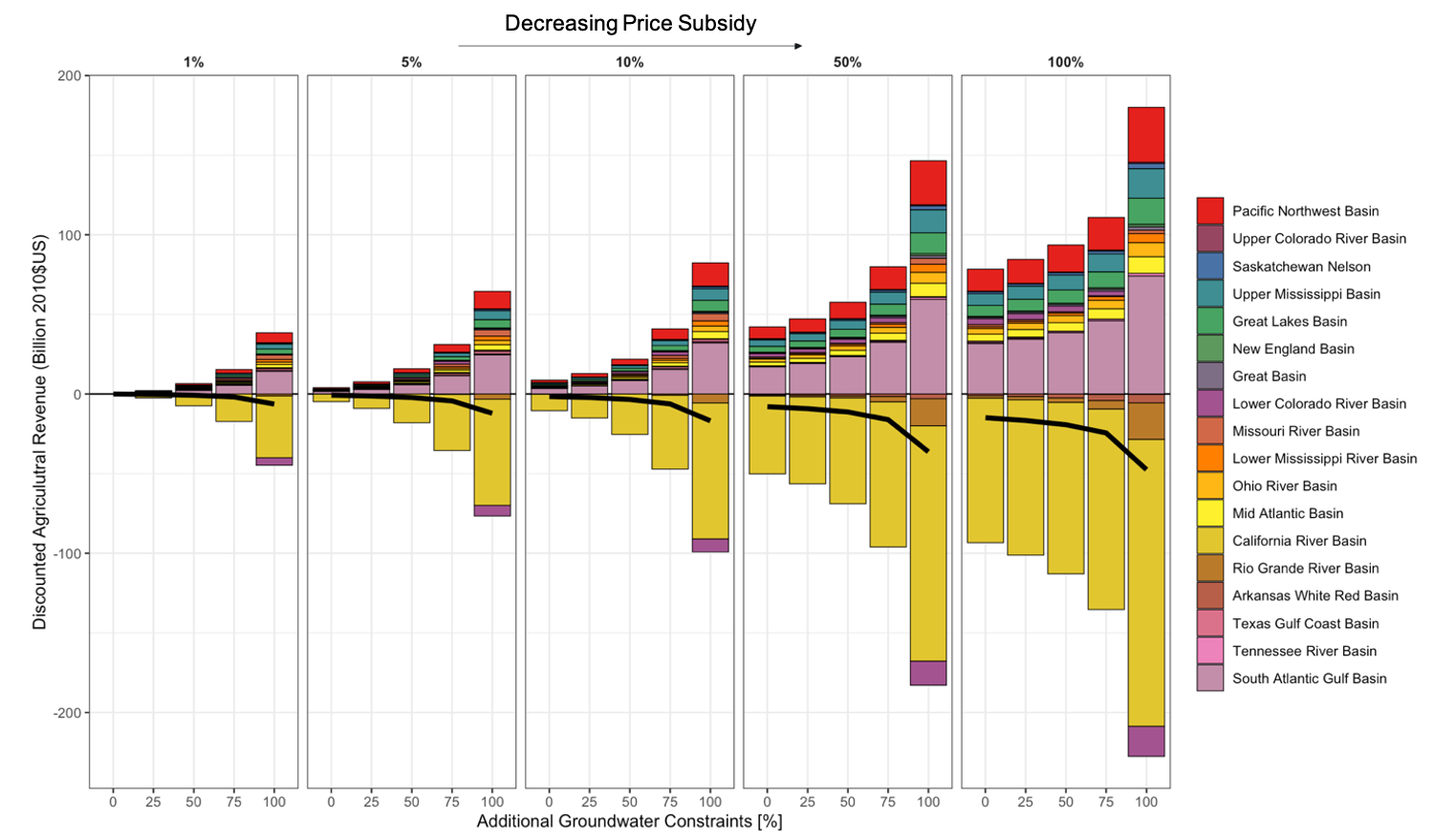


**Supplementary Figure 4. Discounted agricultural revenue for the 2015-2100 time period across all scenarios.** Changes in agricultural revenue from the reference scenario when discounted to 2015 at 5%. Groundwater constraint level is shown on the x-axis while increasing subsidy levels are shown as facets (left-to-right). Basin level revenue impacts are shown as colored bars and United States total change is given as the solid black line. Each Scenario is compared to the reference scenario (0% reduction, 1% subsidy) shown as the left most stack of bars.


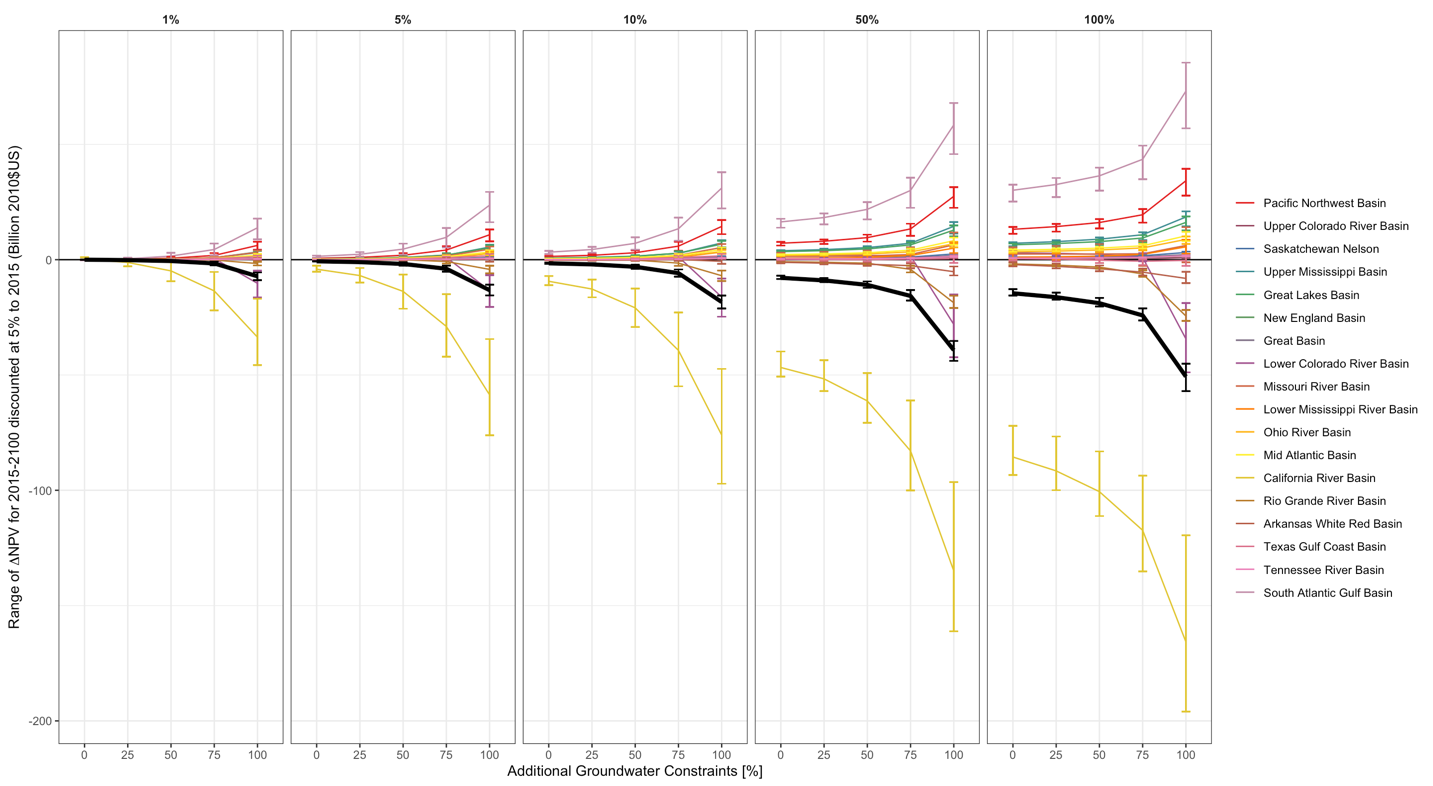


**Supplementary Figure 5. Variability of 2015-2100 cumulative change in agricultural revenue across all scenarios including climatic impacts and desalination availability to water supply.** Variability brought upon GCM-derived water availability for an RCP6.0 scenario and the addition of seawater desalination capabilities where the solid line represents the mean of all scenarios and the total range is shown as the error bars. Variability is shown to be much higher in some basins (such as the California River Basin) than the United States as a whole.

**
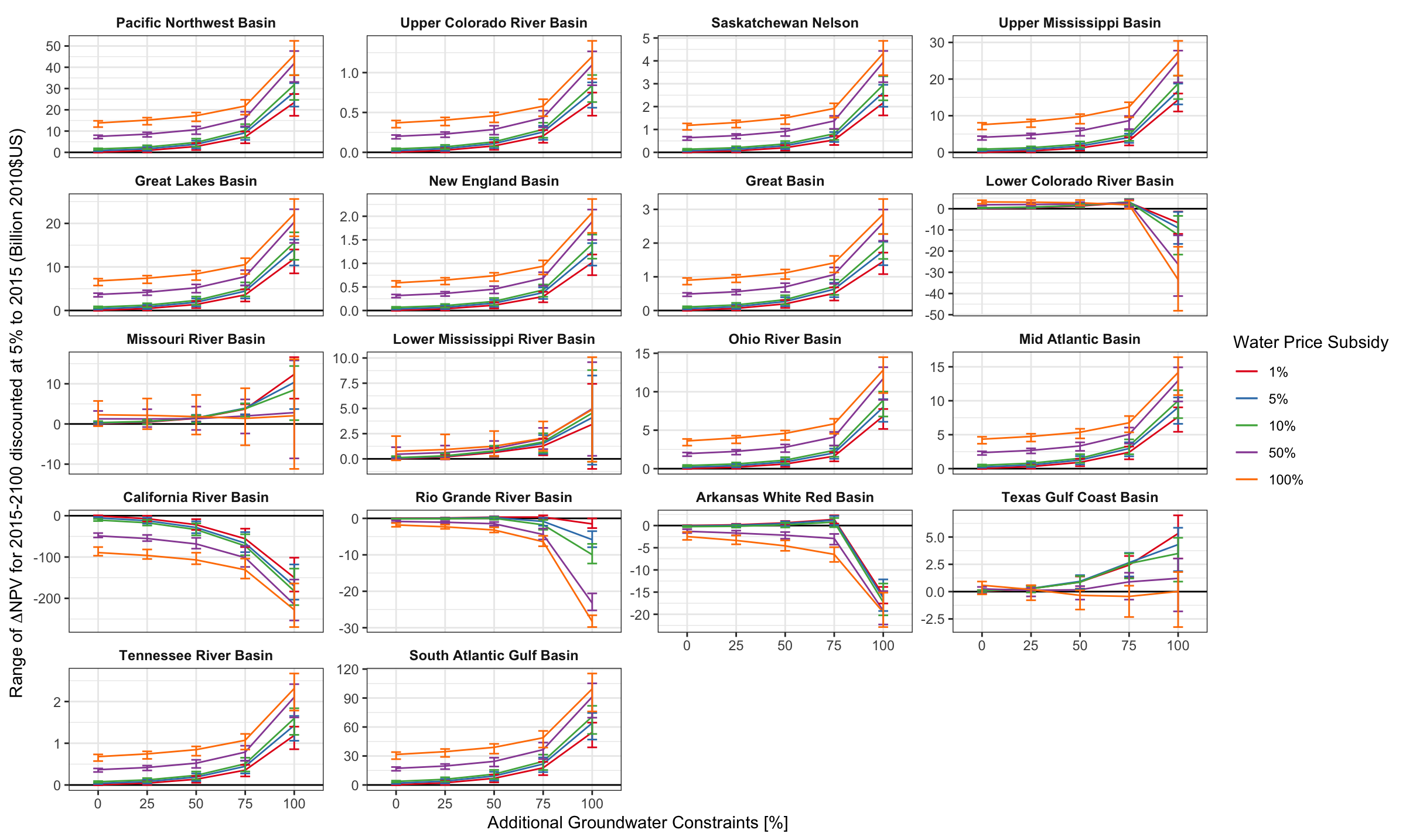
**

**Supplementary Figure 6. Variability of 2015-2100 cumulative change in agricultural revenue across all scenarios including climatic impacts and seawater desalination availability to water supply, by basin.** Variability brought upon GCM-derived water availability for an RCP6.0 scenario and the addition of seawater desalination capabilities where the solid line represents the mean of all scenarios and the total range is shown as the error bars.

**
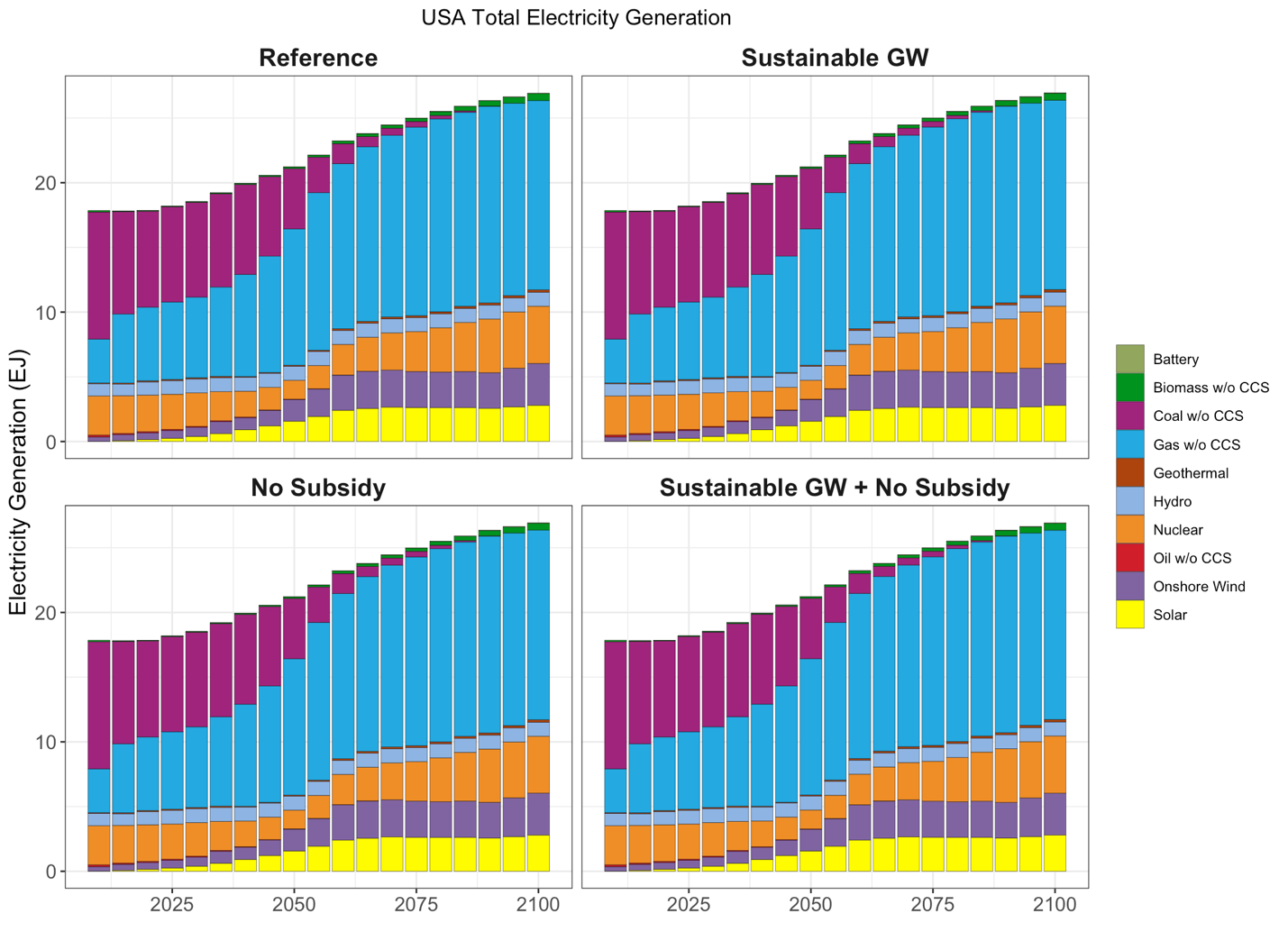
**

**Supplementary Figure 7. Electricity Generation by fuel type in the United States.** No significant differences arise due to the inclusion of sustainable water measures described in Table 1. Results shown for the entire United States.

**
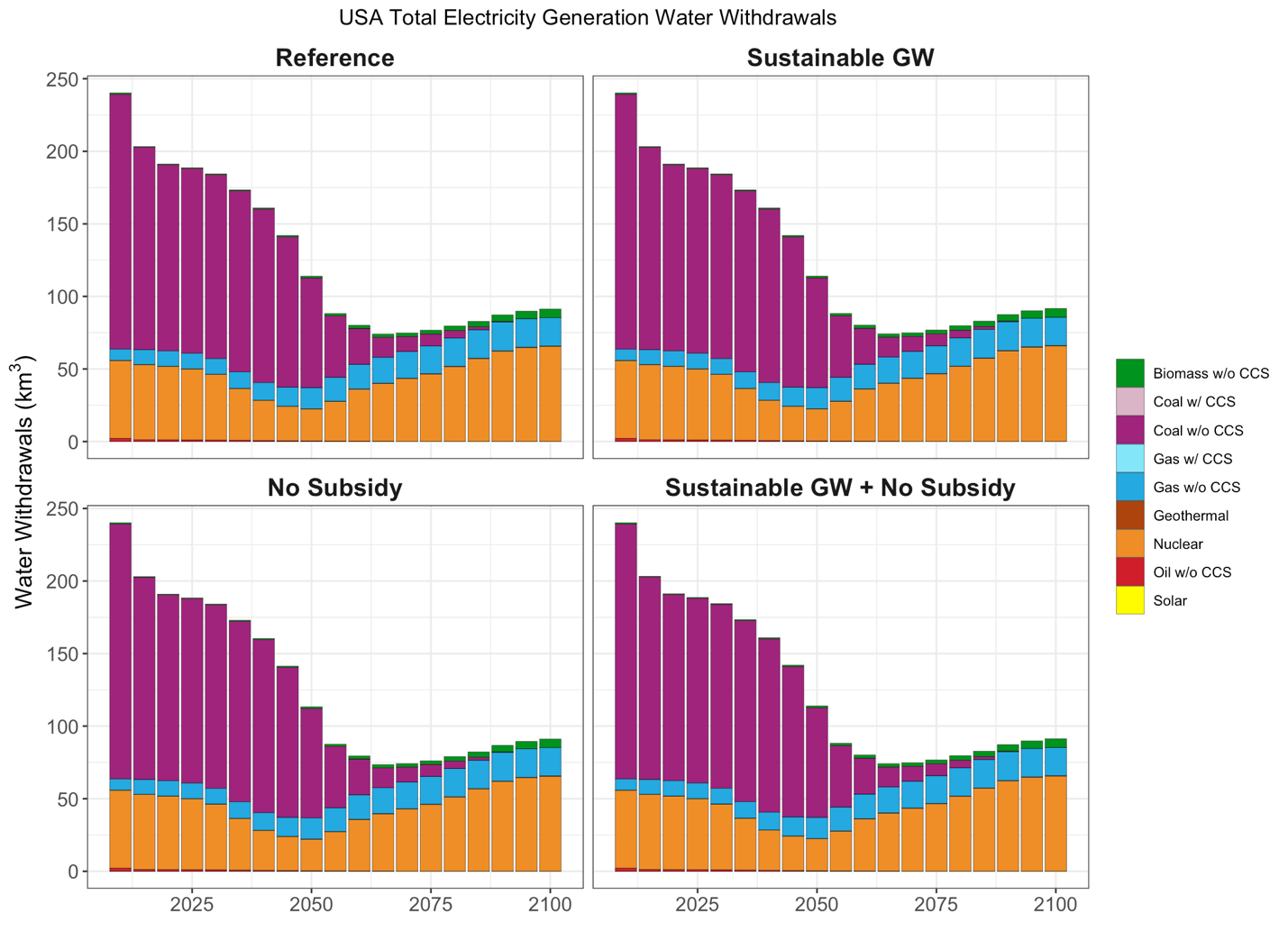
**

**Supplementary Figure 8. Electricity generation water withdrawals by fuel type in the United States.** Results shown for the entire United States region.


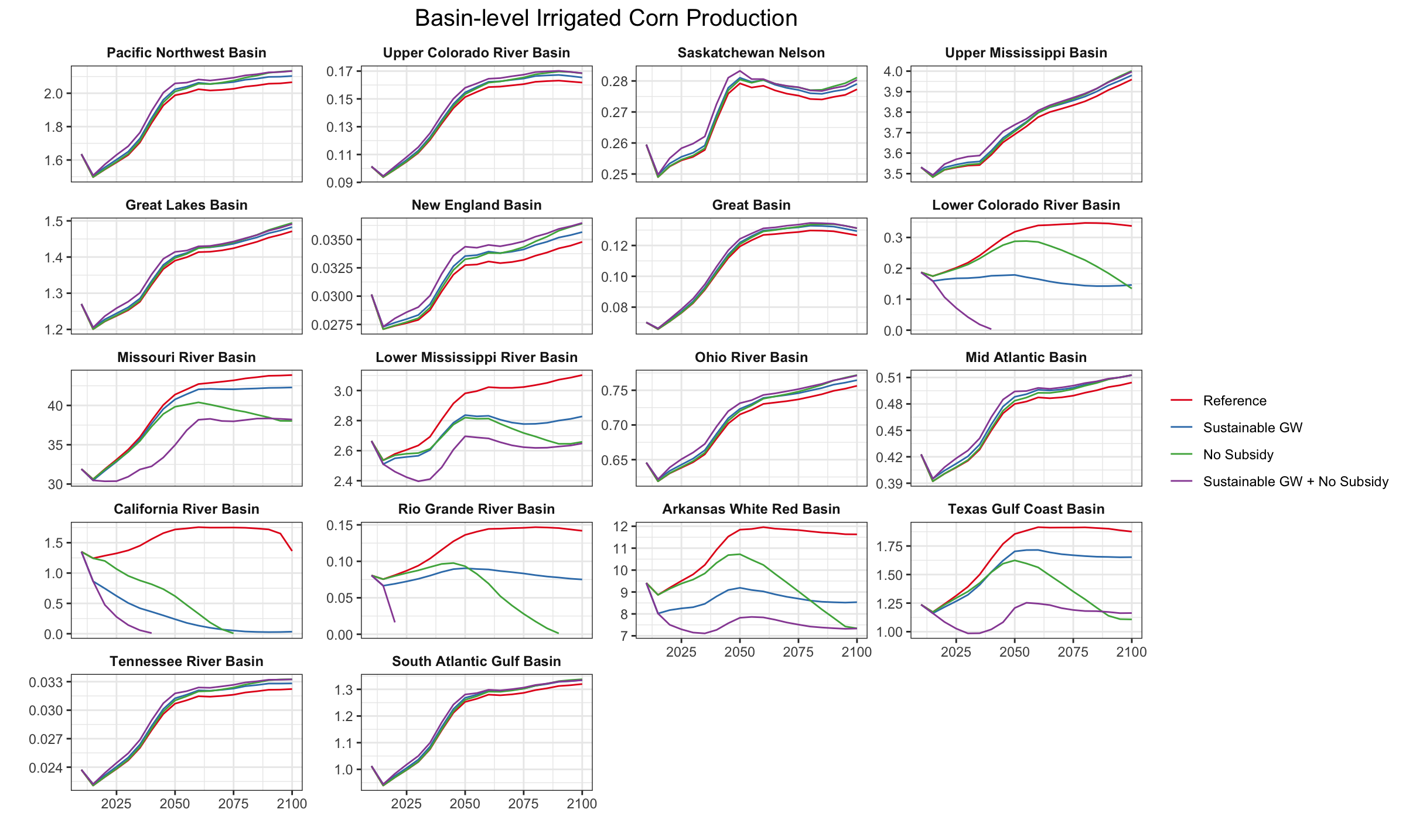


**Supplementary Figure 9. Basin level Irrigated Corn Production (Mt).** Impacts of sustainability scenarios on basin level production of irrigated corn crops in the USA.


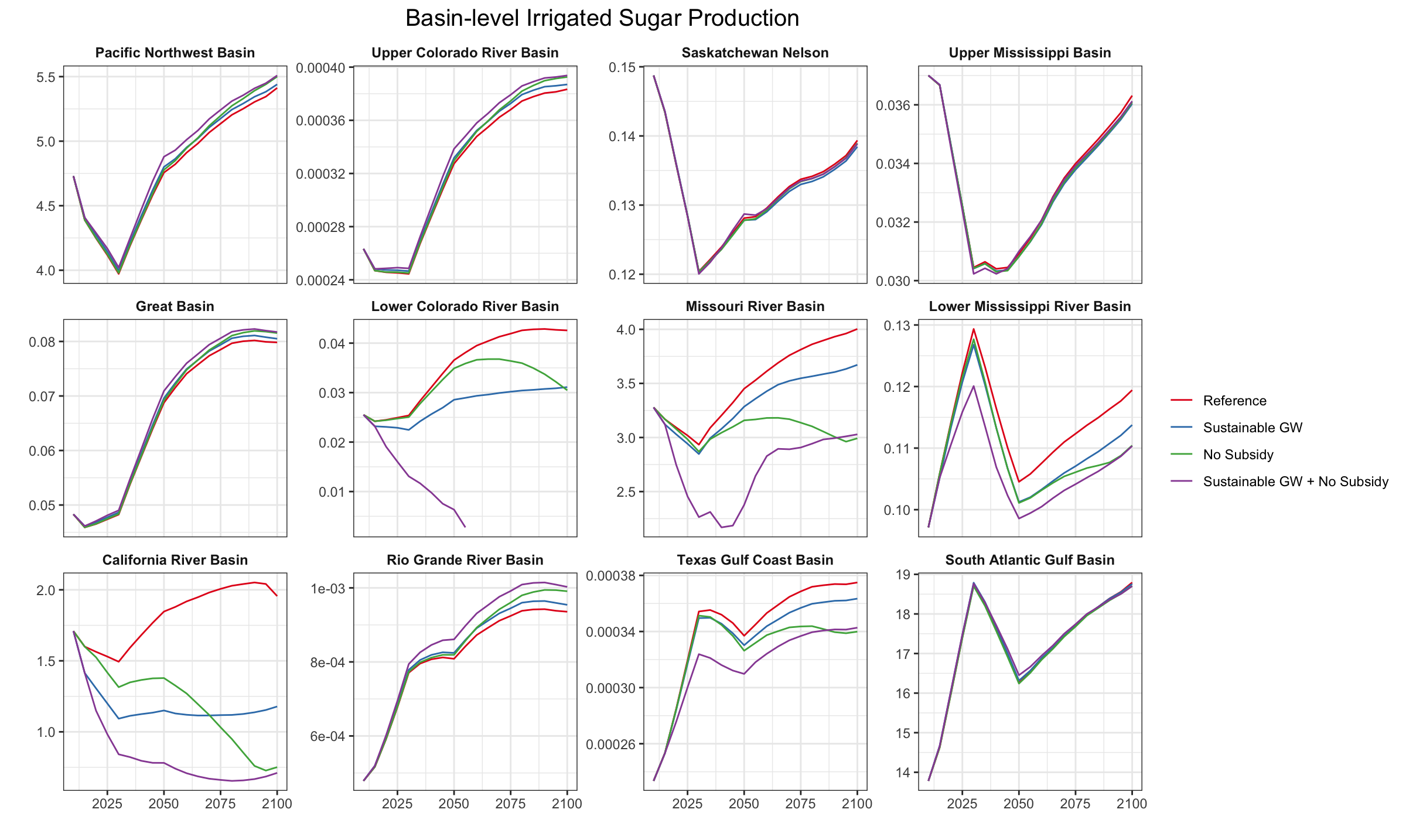


**Supplementary Figure 10. Basin level Irrigated Sugar Production (Mt).** Impacts of sustainability scenarios on basin level production of irrigated sugar crops in the USA.


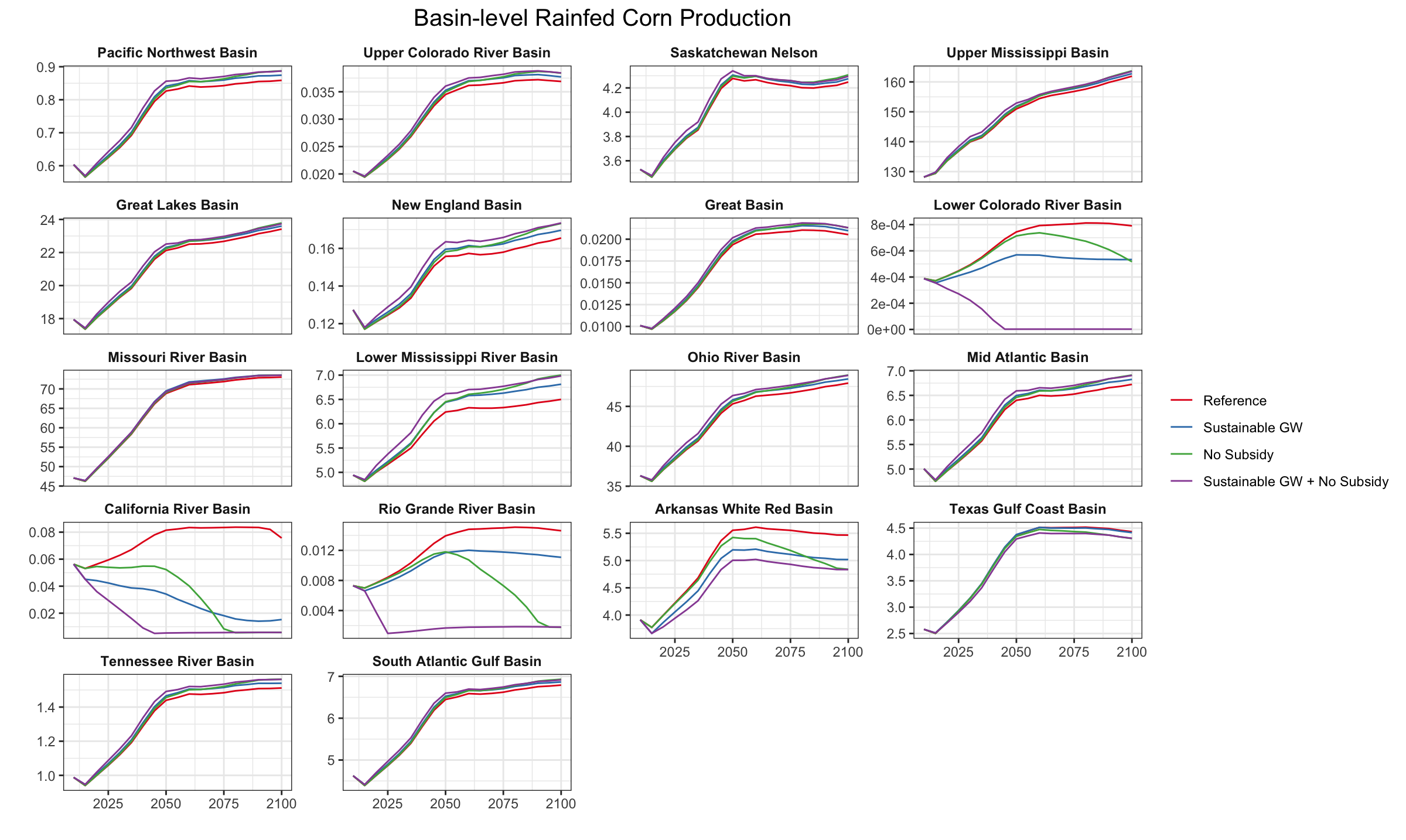


**Supplementary Figure 11. Basin level Rainfed Corn Production (Mt).** Impacts of sustainability scenarios on basin level production of rainfed corn crops in the USA.


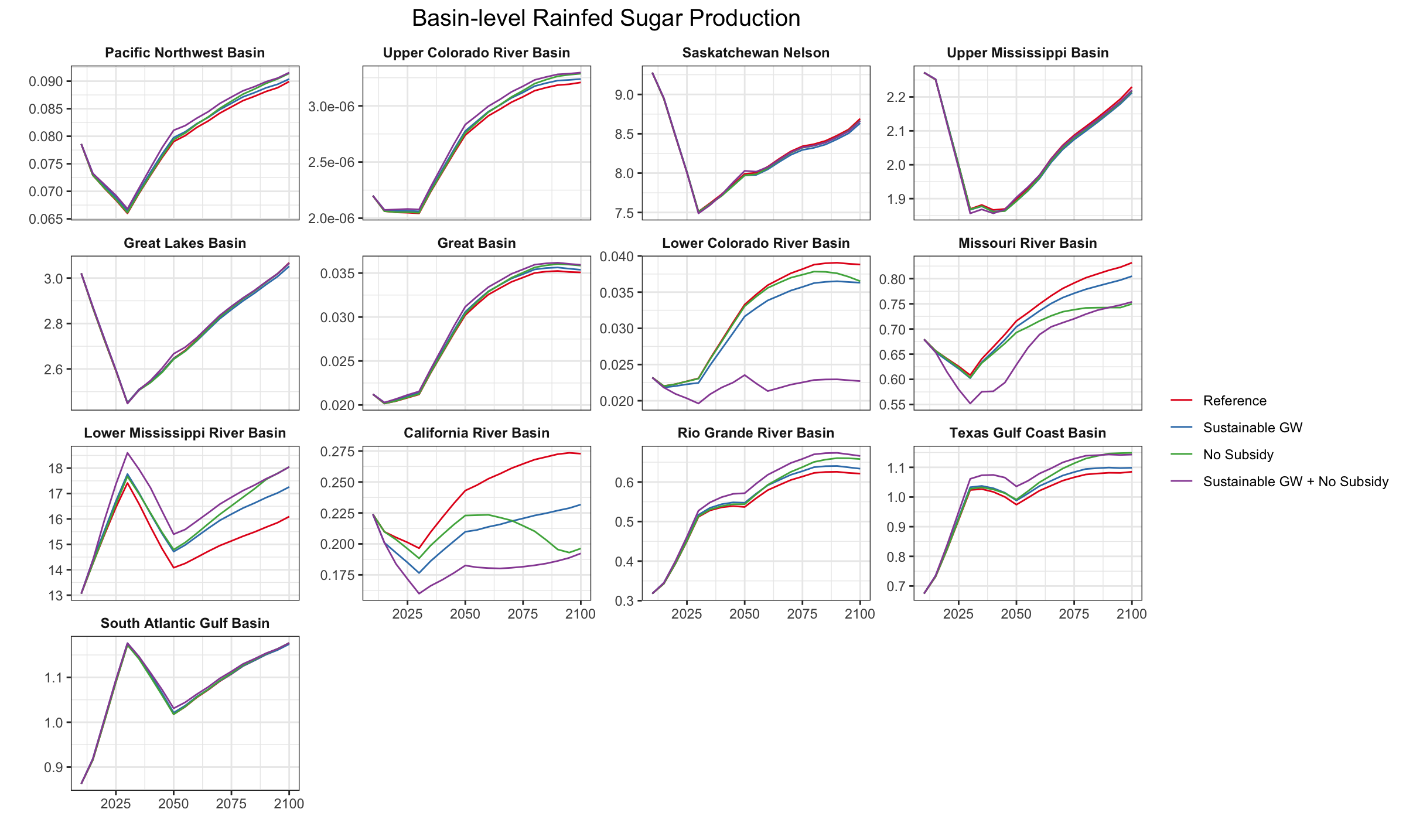


**Supplementary Figure 12. Basin level Rainfed Sugar Production (Mt).** Impacts of sustainability scenarios on basin level production of rainfed sugar crops in the USA.


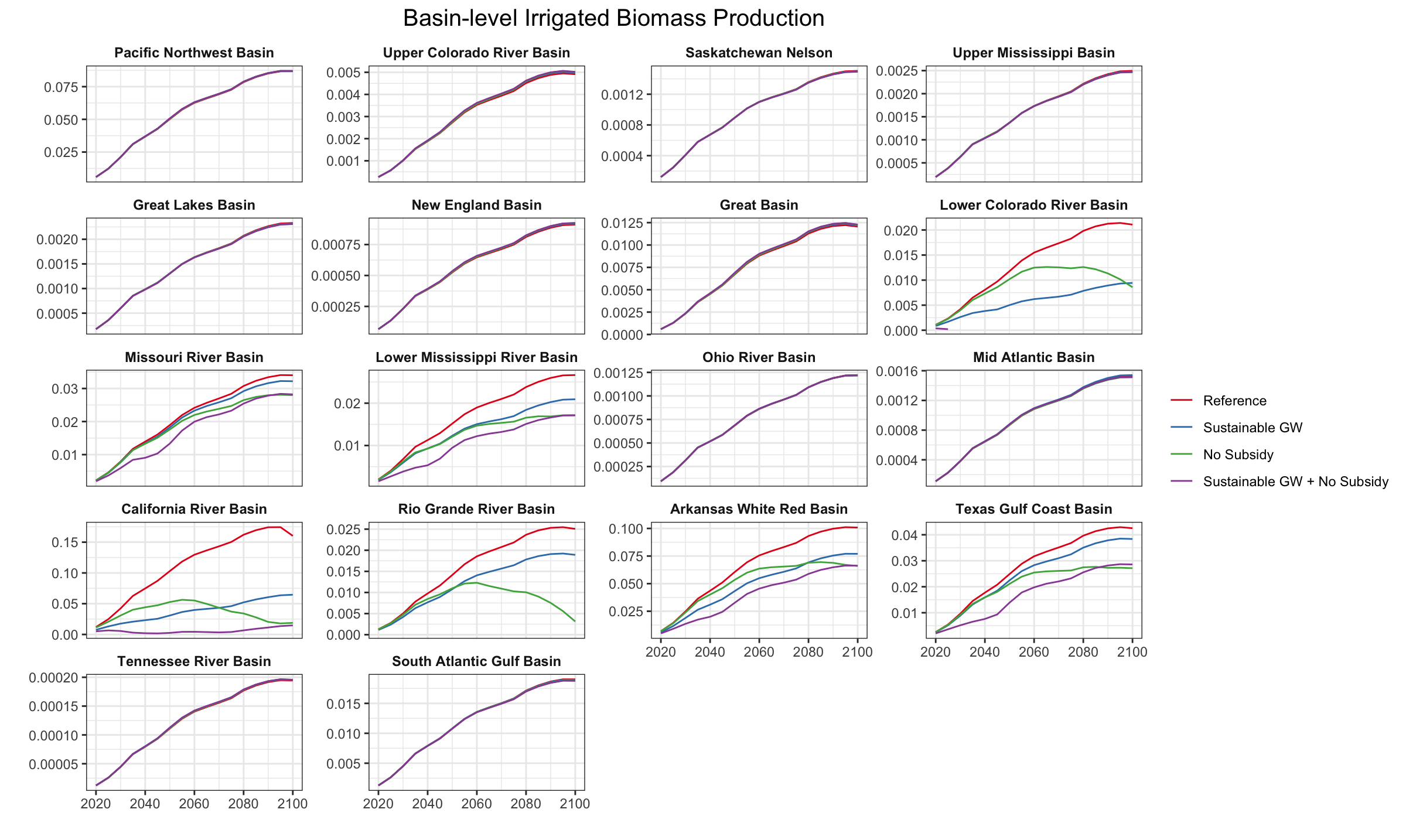


**Supplementary Figure 13. Basin level Irrigated Biomass Production (EJ).** Impacts of sustainability scenarios on basin level production and the energy derived from irrigated biomass crops in the USA.
